# Regulation of Motility and Phenazine Pigment Production by FliA Is Cyclic-di-GMP Dependent in *Pseudomonas aeruginosa* PAO1

**DOI:** 10.1371/journal.pone.0155397

**Published:** 2016-05-13

**Authors:** Yi-Ling Lo, Lunda Shen, Chih-Hsuan Chang, Manish Bhuwan, Cheng-Hsun Chiu, Hwan-You Chang

**Affiliations:** 1 Institute of Molecular Medicine, National Tsing Hua University, Hsin Chu, Taiwan; 2 Molecular Infectious Disease Research Center, Division of Pediatric Infectious Diseases, Department of Pediatrics, Chang Gung Memorial Hospital, Chang Gung University College of Medicine, Taoyuan, Taiwan; Centre National de la Recherche Scientifique, Aix-Marseille Université, FRANCE

## Abstract

The transcription factor FliA, also called sigma 28, is a major regulator of bacterial flagellar biosynthesis genes. Growing evidence suggest that in addition to motility, FliA is involved in controlling numerous bacterial behaviors, even though the underlying regulatory mechanism remains unclear. By using a transcriptional fusion to *gfp* that responds to cyclic (c)-di-GMP, this study revealed a higher c-di-GMP concentration in the *fliA* deletion mutant of *Pseudomonas aeruginosa* than in its wild-type strain PAO1. A comparative analysis of transcriptome profiles of *P*. *aeruginosa* PAO1 and its *fliA* deletion mutant revealed an altered expression of several c-di-GMP-modulating enzyme-encoding genes in the *fliA* deletion mutant. Moreover, the downregulation of *PA4367* (*bifA*), a Glu-Ala-Leu motif-containing phosphodiesterase, in the *fliA* deletion mutant was confirmed using the β-glucuronidase reporter gene assay. FliA also altered pyocyanin and pyorubin production by modulating the c-di-GMP concentration. Complementing the *fliA* mutant strain with *bifA* restored the motility defect and pigment overproduction of the *fliA* mutant. Our results indicate that in addition to regulating flagellar gene transcription, FliA can modulate the c-di-GMP concentration to regulate the swarming motility and phenazine pigment production in *P*. *aeruginosa*.

## Introduction

The transcription factor FliA, also called sigma factor 28, is a major transcription factor regulating flagellar biosynthesis [1,2]. The motility of *fliA* mutants of *Escherichia coli*, *Pseudomonas aeruginosa*, and *Bacillus subtilis* is significantly compromised, whereas the complementation of the *fliA* gene could rescue the defective motility in *fliA* mutants [2,3]. The importance of *fliA* in flagellar biosynthesis and chemotaxis has also been characterized in numerous other bacterial species, including *Pseudomonas putida*, *Vibrio cholerae*, *Campylobacter jejuni*, *Edwardsiella tarda*, and *Yersinia pestis* [4–8].

In addition to controlling flagellar biosynthesis at the transcriptional level, bacterial motility is also posttranslationally regulated. A mechanism independent of flagellar biosynthesis involving YhjH and YcgR proteins was observed in *E*. *coli* [9]. YhjH, a phosphodiesterase (PDE), governs the downregulation of cyclic (c)-di-GMP (bis-(3ʹ-5ʹ)-c-di-GMP) concentrations. YcgR, a c-di-GMP-binding protein, is a key regulator of flagellar motor control, responding to c-di-GMP and inhibiting motility by interacting with the flagellar switch complex proteins FliG and FliM [9,10]. In *E*. *coli*, expression of *yhjH* is found to be dependent on FliA [11].

C-di-GMP is a universal intracellular second messenger in bacteria [12–14]. It plays crucial roles in complex physiological processes, including virulence, motility, biofilm formation, and cell cycle progression [12,14–17]. Diguanylate cyclases (DGCs) and cyclic nucleotide PDEs cause the synthesis and degradation of c-di-GMP, respectively [18]. DGCs typically contain a conserved Gly-Gly-Asp-Glu-Phe (GGDEF) or Gly-Gly-Glu-Glu-Phe (GGEEF) domain, whereas PDEs typically contain a Glu-Ala-Leu (EAL) domain [19,20].

*P*. *aeruginosa* is an opportunistic pathogen possessing a polar flagellum for swimming in a liquid environment and swarming motility on semisolid surfaces [21,22]. *P*. *aeruginosa* also requires flagella to adhere to the host epithelium at the initiation of infection [23,24]. As a global regulator, FliA is considered to regulate the expression of genes involving flagellin production, initiation of flagella filament assembly and length control, chemotaxis regulator, motor rotation, and FliA-specific anti-sigma factor in *P*. *aeruginosa* [22]. Because there is no *E*. *coli yhjH* ortholog present in *P*. *aeruginosa*, whether FliA can regulate motility through c-di-GMP in *P*. *aeruginosa* is not clear. The present study aimed to determine whether FliA can regulate bacterial motility and pigment production through the second messenger c-di-GMP. Our results indicate that intracellular c-di-GMP concentrations are altered in the *fliA* deletion mutant and that a PDE, BifA contributes to the FliA-mediated c-di-GMP regulation and further affects the bacterial physiology.

## Materials and Methods

### Bacterial strains and plasmids

The strains and plasmids of *P*. *aeruginosa* used in this study are listed in Table 1. The bacterial strains were propagated in Luria–Bertani (LB) broth supplemented with an appropriate antibiotic, as indicated in Table 1. The growth rates of the strains were determined through spectrometry by measuring the absorbance at 595 nm or by enumerating the colony-forming units after plating the bacterial cells on LB agar and incubating overnight.

**Table 1:** Bacteria strains and plasmids used in this study.

Strain/plasmid	Relevant characteristics	Sources
***E*. *coli***		
S17-1 λ *pir*	Tp^r^ Sm^r^ *recA*, *thi*, *pro*, *hsdR*^*-*^*M*^*+*^ RP4: 2-Tc::*Mu*:Km^r^ Tn*7* λ*pir*	Laboratory stock
***P*. *aeruginosa***		
PAO1	Non-mucoid wild type strain	Laboratory stock
Δ*fliA*	*fliA* deletion mutant of PAO1	This study
PAO1[pMMB66EH]	PAO1, carrying pMMB66EH, Cb^r^	This study
Δ*fliA* [pMMB66EH]	Δ*fliA*, carrying pMMB66EH, Cb^r^	This study
Δ*fliA*[pMMB2133]	Δ*fliA*, carrying pMMB2133, Cb^r^	This study
Δ*fliA*[pMMB4367]	Δ*fliA*, carrying pMMB4367, Cb^r^	This study
**Plasmids**		
pEX18Tc	Suicide vector; *oriT*^*+*^, *sacB*^*+*^, Tc^r^	Laboratory stock
pMMB66EH	Broad-host-range expression vector, Ap^r^	Laboratory stock
pMMB*fliA*	pMMB66EH containing *fliA*, Ap^r^	This study
pMMB2133	pMMB66EH containing *PA2133*, Ap^r^	Laboratory stock
pMMB4367	pMMB66EH containing *PA4367*, Ap^r^	Laboratory stock
pCHUB237	GUS reporter vector, Gm^r^	Wen-Ling Deng ^a^
pCHUB-P_*PA4367*_	pCHUB237 containing P_*PA4367*_, Gm^r^	This study
pCHUB-P_*PA5017*_	pCHUB237 containing P_*PA5017*_, Gm^r^	This study
pCdrA-*gfp*^C^	c-di-GMP reporter plasmid	Tim Tolker-Nielsen ^b^

^*a*^ National Chung Hsing University, Taiwan

^*b*^ University of Copenhagen, Denmark

### Recombinant DNA techniques

Genomic DNA of *P*. *aeruginosa* was isolated using the Wizard^®^ Genomic DNA Purification kit (Promega). Plasmid DNA was isolated using the Presto^™^ Mini Plasmid kit (Geneaid). The DNA quality was determined using agarose gel electrophoresis, and the DNA concentration was identified using a NanoDrop ND-1000 spectrophotometer. Moreover, DNA was amplified using dNTPs (GeneDirex), *pfu* DNA polymerase (Thermo Scientific), DreamTaq DNA polymerase (Thermo Scientific), an appropriate primer set, and a template DNA. The amplified DNA fragments were then purified using a Gel/PCR DNA fragment extraction kit (Geneaid). Restriction endonucleases were purchased from New England Biolab Inc. and were used according to the manufacturer’s instructions.

### Construction of an isogenic *fliA* mutant and a complementary strain

A *P*. *aeruginosa fliA* deletion mutant was constructed using homologous recombination. The 5ʹ and 3ʹ regions (approximately 1 kb in length) flanking *fliA* were cloned into the suicide plasmid pEX18Tc. The resulting plasmid was transformed into *E*. *coli* S17-1 λ *pir* and mobilized into *P*. *aeruginosa* PAO1 through conjugation. The first crossover recombinants were selected on LB agar supplemented with 100 μg/ml tetracycline (Sigma), and the second crossover strains were selected on LB agar supplemented with 10% sucrose (J. T. Baker). These strains were further analyzed using PCR for determining *fliA* deletion. The *fliA* complementation strains were constructed using pMMB66EH-based plasmids.

### Quantification of the phenazine pigment production

*P*. *aeruginosa* strains were propagated in a 5-ml Pseudomonas broth (PB) containing 2% (w/v) Bacto Peptone (Difco), 0.14% (w/v) MgCl_2_ (J. T. Baker), 1% (w/v) K_2_SO_4_ (Sigma), and 500 μg/ml carbenicillin (Sigma) and were incubated at 37°C with shaking at 150 rpm for 16 h. The pigments in the bacterial culture were extracted with chloroform (Sigma). After centrifugation, the chloroform layer was mixed with 1 ml of 0.2 N HCl (Sigma), which yielded a pink solution, and the absorbance was measured at OD_520_. The bacterial cell number was normalized using the cell density (OD_595_) of the culture to reflect pyocyanin production per cell. Similarly, the OD_520_ of the aqueous phase was also measured and normalized to indicate pyorubin production per cell.

### Transcriptome analysis

The bacterial cells on the periphery (5 mm) of the swarming bacterial mass were collected, and RNA of that was extracted using the Trizol reagent (Invitrogen) and purified using the PureLink^™^ RNA Mini kit (Ambion). Furthermore, the RNA quality was examined using agarose gel electrophoresis and was quantified using a NanoDrop ND-1000 spectrophotometer. For RNA sequencing, the RNA was reverse transcribed into complementary DNA and then analyzed using HiSeq 2000 (Illumina, Inc.), producing approximately 40.7 million and 44.6 million 100-bp pair-end reads from PAO1 [pMMB66EH] and Δ*fliA* [pMMB66EH] corresponding to coverage rates of 650.35- and 712.20- fold, respectively.

### Bioinformatics analysis

The genomic sequence of *P*. *aeruginosa* PAO1 was obtained from the *Pseudomonas* genome database (www.pseudomonas.com) and Kyoto Encyclopedia of Genes and Genomes (www.genome.jp/kegg/). Nucleotide and protein sequences were compared using BLASTn and BLASTp, respectively; both programs were provided by the National Center for Biotechnology Information (www.ncbi.nlm.nih.gov). Restriction sites and oligonucleotide primer sequences were identified using Vector NTI Advance^®^ software (Invitrogen). Promoter regions were predicted using BPROM software (Softberry, Inc).

### Flagellar observation through transmission electron microscopy

Overnight cultures of *P*. *aeruginosa* PAO1 [pMMB66EH], Δ*fliA* [pMMB66EH], and PAO1 [pMMB*fliA*] were diluted 100 folds with fresh LB broth and were negatively stained with 0.1% (w/v) uranyl acetate (Electron Microscopy Sciences, Hatfield, PA, USA). Flagellar morphologies were observed using a transmission electron microscope (Hitachi HT7700).

### Swarming and swimming motility assay

The swarming assay plates contained 0.5% (w/v) glucose (Amersco), 0.5% (w/v) agar (Amersco), and 0.8% (w/v) nutrient broth containing 3.0 g/L beef extract (BD) and 5.0 g/L peptone (BD), supplemented with 0.5 mM isopropyl-β-d-thiogalactopyranoside (IPTG; Amersco) and 500 μg/ml carbenicillin (Sigma). Moreover, 3 μl of overnight liquid cultures of each test strain were inoculated on a swarming plate. After the plate was incubated at 37°C for 24 h, the longest swarming distance of the cells was measured. The swimming assay plates contained 1.0% (w/v) tryptone (Scharlab), 0.3% (w/v) agar (Amersco), and 1.0% (w/v) NaCl (J.T. Baker), supplemented with 500 μg/ml carbenicillin and 0.5 mM IPTG. Moreover, 3 μl of overnight liquid culture of each strain was inoculated on the plate and incubated at 37°C for 24 h; the diameters of the swimming zone were then measured.

### Cyclic-di-GMP measurement

The c-di-GMP reporter assay was performed using pCdrA–*gfp*^C^, a plasmid carrying the c-di-GMP responsive promoter of the *cdrA* gene fused with a green fluorescence-encoding gene [25]. The plasmid was introduced into *P*. *aeruginosa* wild-type PAO1 and its Δ*fliA* mutant, followed by growing the transformants under three conditions: in LB broth and on swarming and swimming plates. These pCdrA–*gfp*^C^-carrying strains were grown in LB broth supplemented with 60 μg/ml gentamycin (Amersco) at 37°C with overnight shaking at 150 rpm. The cells from 1 ml of overnight culture of each strain were collected by centrifugation at 5000 *g* for 3 min and were resuspended in sterile phosphate-buffered saline (PBS). The cell density at OD_595_ was adjusted to 0.3, and 100 μl of the cell suspension was added to each well in a black 96-well microtiter plate. The fluorescence intensity was then measured using excitation and emission wavelengths of 515 nm and 490 nm in a luminometer (Wallac 1420 Victor^2^). Furthermore, the c-di-GMP reporter assay was performed on plates, and the green fluorescence of the cells was detected using an upright epifluorescence microscope (BX-51; Olympus) equipped with a digital camera. Bright-field and fluorescence images obtained through microscopy were merged and analyzed using SPOT Advanced plus Imaging Software 4.6 (Spot Imaging, Inc.). Similarly, cells at the periphery (approximately 5 mm) of the swarming area were collected and resuspended in PBS, the green fluorescence intensity was measured, and the values were normalized with the cell density.

### β-glucuronidase reporter gene assay

The promoter activity assay was performed using pCHUB238, a plasmid carrying a β-glucuronidase (GUS) reporter gene. The GUS reporter enzyme converts the colorless and nonfluorescent substrate 4-methylumbelliferyl-β-D-glucuronide (MUG) to methylumbelliferone, which absorbs light at 365 nm and emits fluorescence at 455 nm [26]. The promoter region of the target gene was cloned at the 5ʹ end of the GUS reporter gene, and the resulting plasmid was transformed into *E*. *coli* S17-1 λ *pir* and mobilized into *P*. *aeruginosa* through conjugation. Moreover, 20 μl of overnight liquid culture carrying a reporter vector were inoculated into 2 ml of the LB broth supplemented with 10 μg/ml gentamycin and were allowed to grow for 8 h. Subsequently, 1 ml of the culture was transferred into a clear tube and centrifuged at 12,000 *g* for 2 min. The supernatant was discarded, and the pellet was mixed with 1 ml GUS buffer (50 mM sodium phosphate, 10 mM β-mercaptoethanol, 10 mM EDTA, 0.1% sodium lauryl sarcosine, and 0.1% Triton X-100, pH 7.0) by vortexing. This solution was maintained on ice for 20 min. The sample was centrifuged at 12,000 *g* and maintained at 4°C for 3 min, and 25 μl of the supernatant was transferred into each well of a black 96-well microtiter plate containing 50 μl of 3 mM 4-MUG. After incubation at 37°C for 10 min, the reaction was terminated by adding 25 μl of 0.72 M Na_2_CO_3_, and the emission intensity at 455 nm under 365 nm excitation was measured using a Wallac 1420 Victor^2^ luminometer. The protein concentration was determined using the Bradford method for normalizing the GUS results.

## Results

### FliA affects bacterial motility and phenazine pigment production

**Fig 1:**
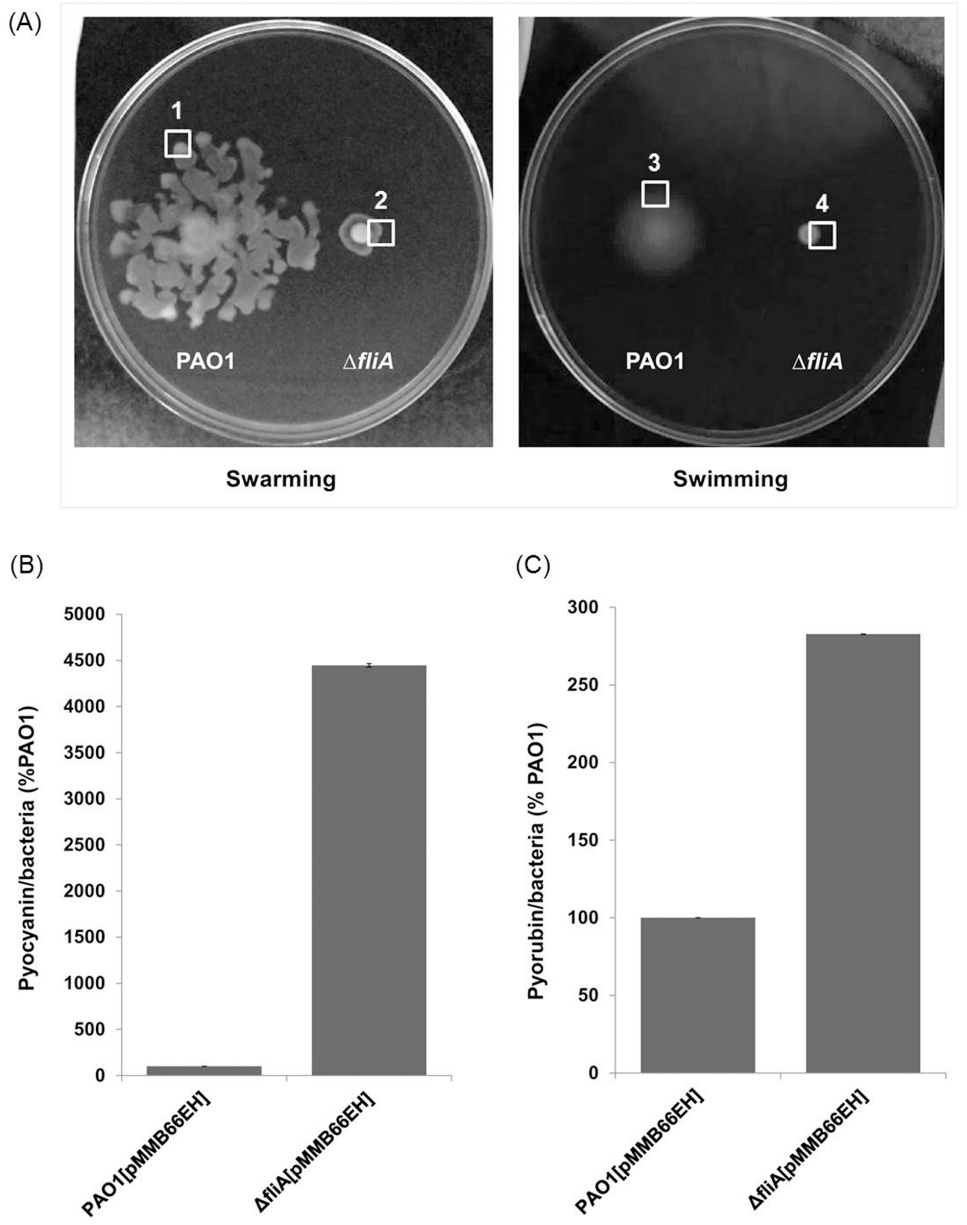
Motility and phenazine pigment production in *P*. *aeruginosa* wild-type PAO1 and *fliA* mutant. (A) Swarming and swimming motility, (B) Pyocyanin production, and (C) Pyorubin production.

To investigate the physiological role of FliA in *P*. *aeruginosa*, we first generated an isogenic *fliA* deletion mutant in *P*. *aeruginosa* PAO1. The motility of the *fliA* mutant was examined on a swarming agar and compared with that of *P*. *aeruginosa* PAO1. The *fliA* deletion mutant exhibited a significant reduction in swarming motility (Fig 1A), which was consistent with the motility of the expected phenotypes [11]. Among the several other biological properties examined, the production of pyocyanin and pyorubin, which are phenazine pigments associated with oxidative stress-mediated cytotoxicity of mammalian cells [27,28], was altered in the *fliA* mutant, demonstrating a 40- and 3-fold enhancement, respectively (Fig 1B and 1C) after 36-h growth in the PB broth.

### FliA affects intracellular c-di-GMP concentrations

**Fig 2:**
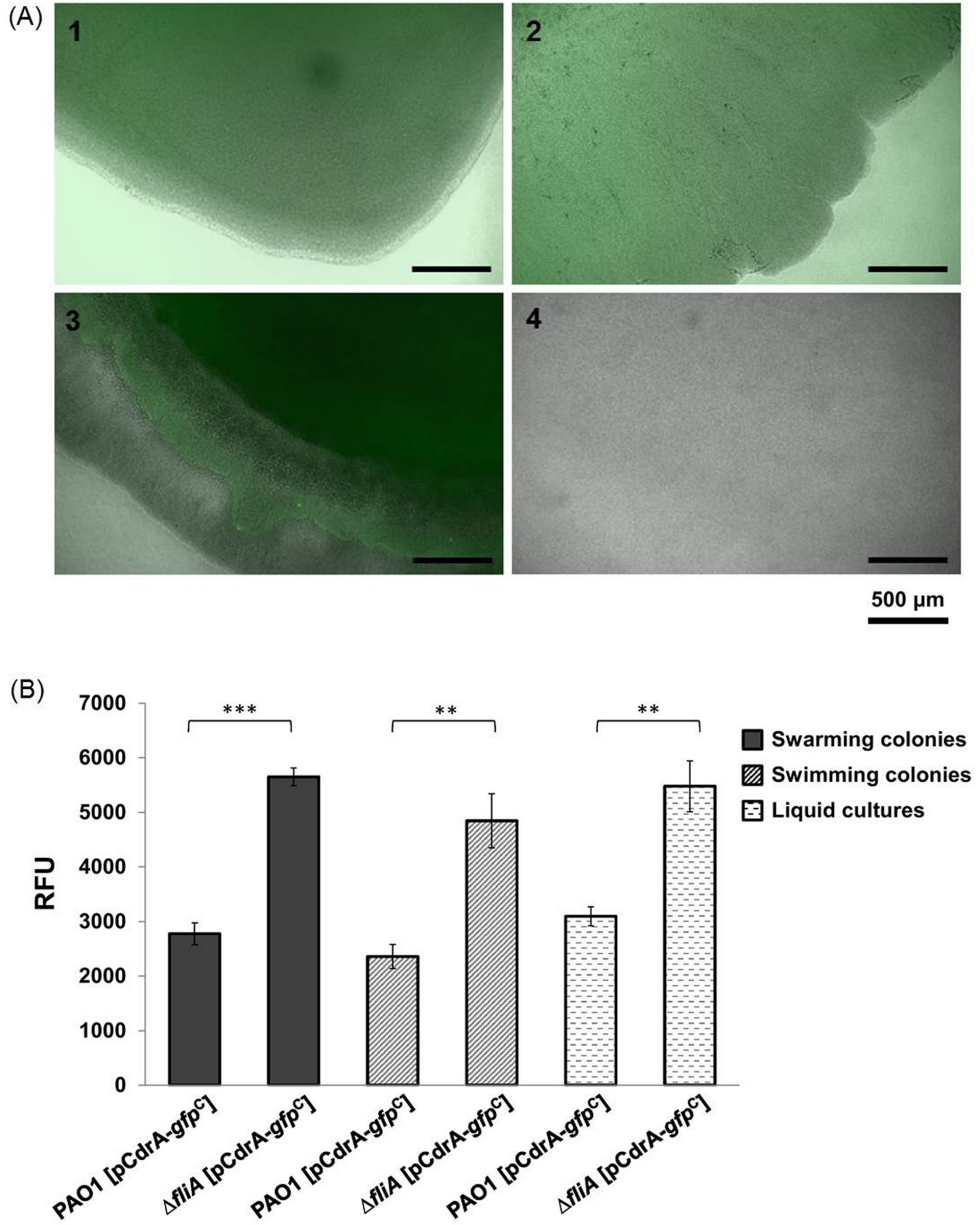
Intracellular concentration of c-di-GMP in *P*. *aeruginosa* wild-type PAO1 and *fliA* mutant. (A) Fluorescence intensity of cells on the periphery of swarming (Panels 1 and 2) and swimming (Panels 3 and 4) areas are presented. Panels 1 and 3, PAO1 [pCdrA–*gfp*C]; 2 and 4, Δ*fliA* [pCdrA–*gfp*C]. (B) Relative fluorescence units of PAO1 [pCdrA–*gfp*^C^] and Δ*fliA* [pCdrA–*gfp*^C^] under different growth conditions.

It has been shown previously that c-di-GMP is a regulator of pyocyanin production [29]. The increase in pyocyanin production in the *fliA* deletion mutant suggested that FliA could affect intracellular c-di-GMP concentrations in *P*. *aeruginosa*. This finding, together with a previous report that FliA can modulate expression of a c-di-GMP metabolizing gene *yhjH* in *E*. *coli* [11], prompted us to investigate whether c-di-GMP participates in the FliA regulon in *P*. *aeruginosa*. This study first measured the effects of *fliA* deletion on intracellular c-di-GMP concentrations by using a c-di-GMP reporter system pCdrA–*gfp*^C^. The fluorescence intensity of both wild-type and *fliA* deletion mutant grown overnight under different conditions, namely in LB broth and on swarming and swimming assay plates, were examined through fluorescence microscopy. Fig 2 shows that the fluorescence intensity of the Δ*fliA* mutant was significantly higher than that of PAO1 particularly in swimming condition, indicating a higher intracellular c-di-GMP concentration in the strain. Further quantitation of the fluorescence intensity using a fluorometer revealed that the fluorescence intensity of PAO1 [pCdrA–*gfp*^C^] was significantly lower than that of Δ*fliA* [pCdrA–*gfp*^C^] under the three examined growth conditions, suggesting that FliA modulates intracellular c-di-GMP concentrations.

### Effects of *fliA* deletion on the expression of c-di-GMP-metabolizing enzyme-encoding genes

This study used RNAseq technology for investigating whether *fliA* deletion alters the expression of genes that encode c-di-GMP-metabolizing enzymes. Total RNAs isolated from *P*. *aeruginosa* strains grown under swarming conditions were reverse transcribed into cDNAs and the transcription profiles of PAO1 and Δ*fliA* strains were compared (NCBI Accession SRP071734), and several EAL domain-containing PDE-coding genes exhibiting moderate differences in expression levels were identified (Table 2). The PDE genes *PA2133*, *PA3311*, *PA5017*, *PA4108*, and *PA4367* demonstrated lower expression levels in the Δ*fliA* strain.

**Table 2:** Expression levels of PDE/DGC-related genes based on the transcriptome analysis of *ΔfliA* compared with that of PAO1.

Gene locus	Gene name	Gene product or function	Fold change
*PA2133*	*-*	PDE	-2.21
*PA3311*	*nbdA*	NbdA, PDE	-1.61
*PA5017*	*dipA*	DipA, PDE	-1.56
*PA4108*	*-*	PDE	-1.40
*PA4367*	*bifA*	BifA, PDE	-1.39
*PA3825*	*-*	Catalytically active PDE	-1.23
*PA2567*	*-*	Catalytically active PDE	-1.16
*PA4781*	*-*	PDE	1.85
*PA0861*	*rbdA*	RbdA, DGC	-1.05
*PA1107*	*roeA*	RoeA, DGC	-1.61
*PA0285*	*-*	Predicted DGC	-2.19
*PA5487*	*-*	DGC	1.33

### FliA regulates the PDE-encoding gene *PA4367*

**Fig 3:**
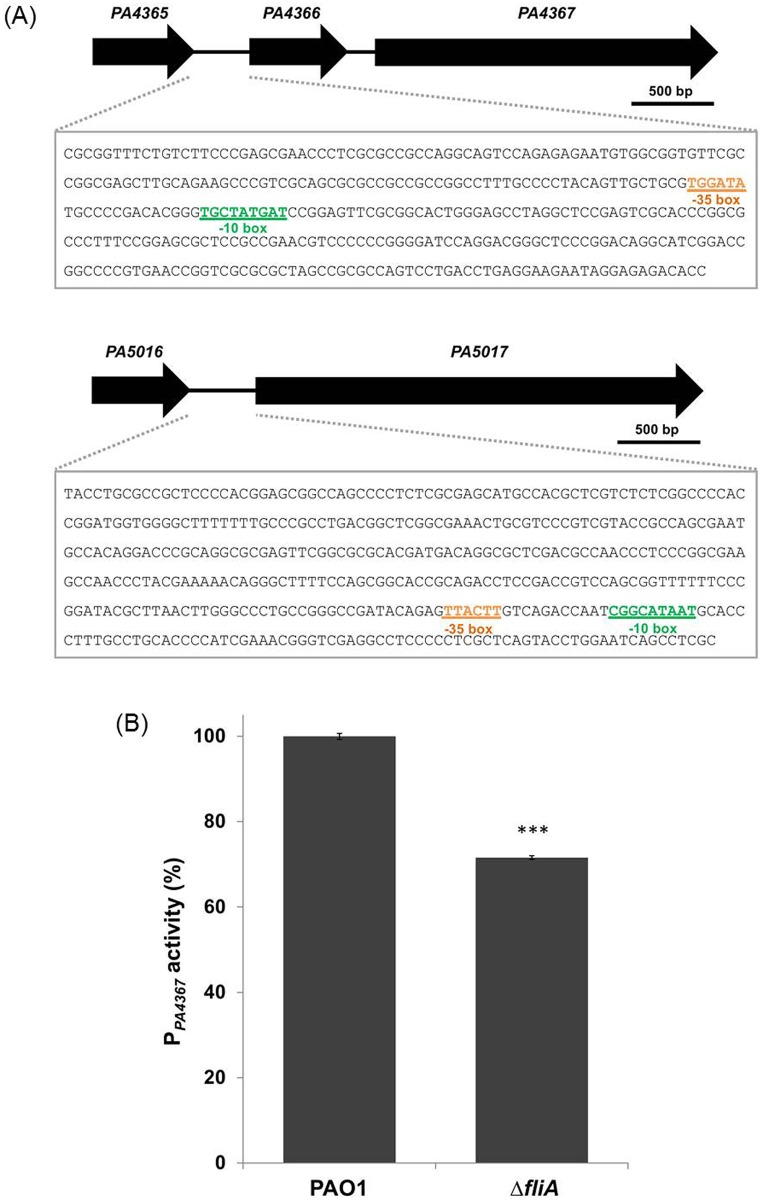
Promoter activity of PDE-coding genes in *P*. *aeruginosa* wild-type PAO1 and *fliA* mutant strains. (A) Promoters of *PA4367* and *PA5017* were predicted using the BPROM program. The DNA sequence of the predicted promoter regions, which were used in the GUS reporter assay, are shown in the block. The possible sigma factor binding sites are also indicated. (B) The promoter activity of *PA4367* in wild-type PAO1 and Δ*fliA* is presented. All experiments were conducted in triplicate, and data are represented as mean ± standard error (*n* = 8; ***, *p* < 0.001).

Among the PDE genes with a lower expression level in the Δ*fliA*, we further analyzed *PA4367* [30] and *PA5017* [31], the two most effectively characterized genes of the group. To investigate whether these two genes were directly regulated by FliA, we analyzed the promoter activities of both genes in the Δ*fliA* and wild-type PAO1 by using the GUS gene reporter system. We employed the bioinformatics tool BPROM to identify the possible promoter regions of *PA4367* and *PA5017* (Fig 3A). We then cloned the DNA fragment comprising the predicted promoters of *PA4367* and *PA5017* into pCHUB238, a plasmid carrying a GUS reporter gene. The resulting plasmids were individually introduced into wild-type PAO1 and the Δ*fliA* mutant, and the GUS activity of the transconjugants was determined. The results revealed that the GUS activity of P_*PA4367*_, but not that of P_*PA5017*_ (data not shown), significantly decreased in the Δ*fliA* mutant (Fig 3B). These findings strongly suggest that *PA4367* is positively regulated by FliA. Nevertheless, the putative FliA binding sequence (TAAAGTTT-N_11_-GCCGATAA) in *Salmonella typhimurium* [32] could not be found in the 500-bp upstream region of *PA4367*. The lack of this canonical FliA promoter sequence suggests that this regulation may be indirect.

### *PA4367* overexpression in the *fliA* mutant rescues the defect in swarming motility and restores phenazine pigment production to normal levels

**Fig 4:**
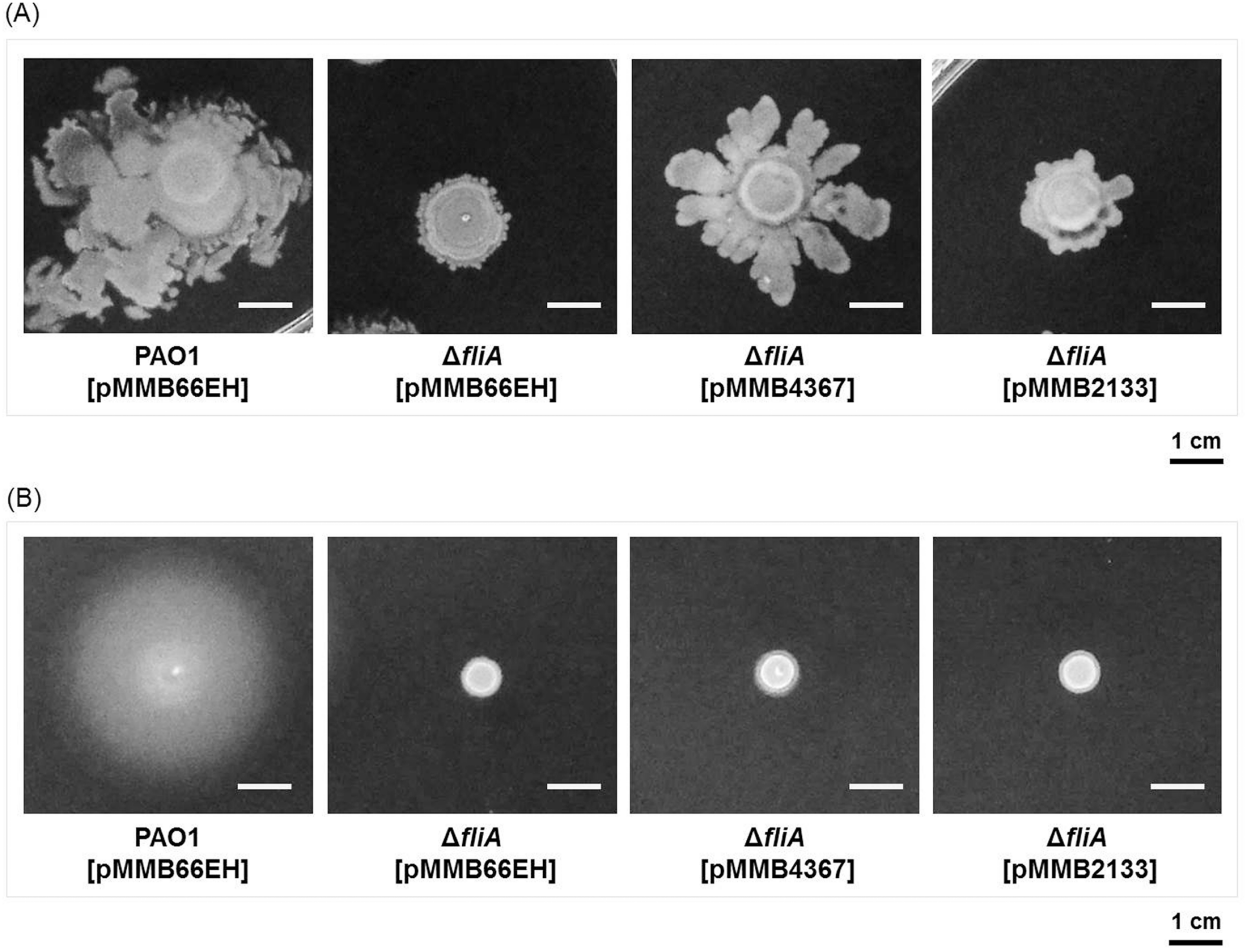
Effects of the c-di-GMP PDE-coding genes *PA4367* and *PA2133* on the swarming motility of the *fliA* mutant. Bacterial culture of each strain were inoculated on the swarming and swimming plates and incubated at 37°C for 24 h. Images of the swarming (A) and swimming (B) pattern of the bacterial strains are presented.

To investigate whether the differences in the swarming motility and phenazine pigment production in the Δ*fliA* mutant were mediated by increases in intracellular c-di-GMP concentrations, we complemented the mutant with a PDE-encoding gene and examined the phenotypes of the complement strain. The deletion of the BifA-coding gene *PA4367* in *P*. *aeruginosa* resulted in a phenotype defective in swarming motility [30], whereas *PA2133* overexpression in *P*. *aeruginosa* reduced the c-di-GMP concentration and impaired biofilm formation [33]. On the basis of these findings, we complemented *PA4367* and *PA2133* in the Δ*fliA* strain and examined their motility. The results revealed that *PA2133* overexpression partly rescued the swarming motility of the Δ*fliA* mutant, whereas *PA4367* complementation restored the swarming motility nearly to the degree of the wild-type strain (Fig 4A). The swimming motility of the *ΔfliA* mutant was not rescued by *PA2133* or *PA4367* overexpression (Fig 4B). The finding further suggests that *fliA* influences the swarming motility but not the swimming motility by modulating the c-di-GMP concentration.

### FliA affects the production of phenazine pigments by modulating the intracellular concentration of c-di-GMP

**Fig 5:**
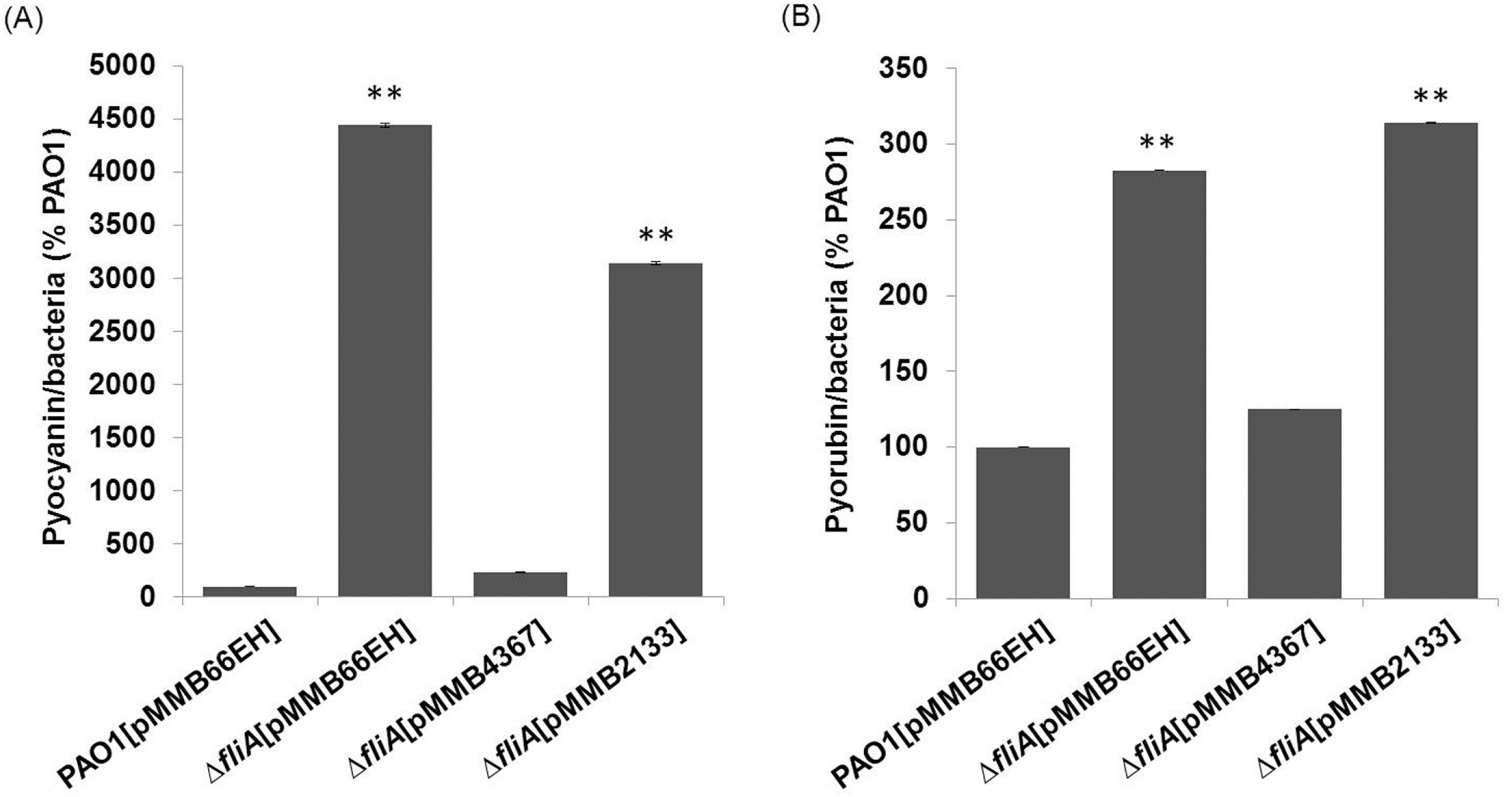
Effects of *fliA* and the c-di-GMP PDE-coding genes *PA4367* and *PA2133* on phenazine pigment production. Pyocyanin (A) and pyorubin (B) produced by different *P*. *aeruginosa* strains were quantified after the cells were grown in a PB medium for 36 h. All experiments were conducted in triplicate, and data are represented as mean ± standard error (*n* = 3; **, *p* < 0.01).

We further investigated whether c-di-GMP is involved in the production of phenazine pigments in the Δ*fliA* mutant. In this study, both *PA4367*- and *PA2133*-overexpressing strains were examined for their production of phenazine pigments of *P*. *aeruginosa*. Introducing functional *PA4367*, but not *PA2133*, into the Δ*fliA* mutant restored the overproduction of pyocyanin and pyorubin phenotype to a concentration comparable with that of the wild-type strain (Fig 5). This finding suggests that *fliA* regulates pyocyanin and pyorubin production through a c-di-GMP-dependent pathway.

### FliA affects swarming motility not only by controlling flagellar biosynthesis but also by modulating the c-di-GMP concentration

**Fig 6:**
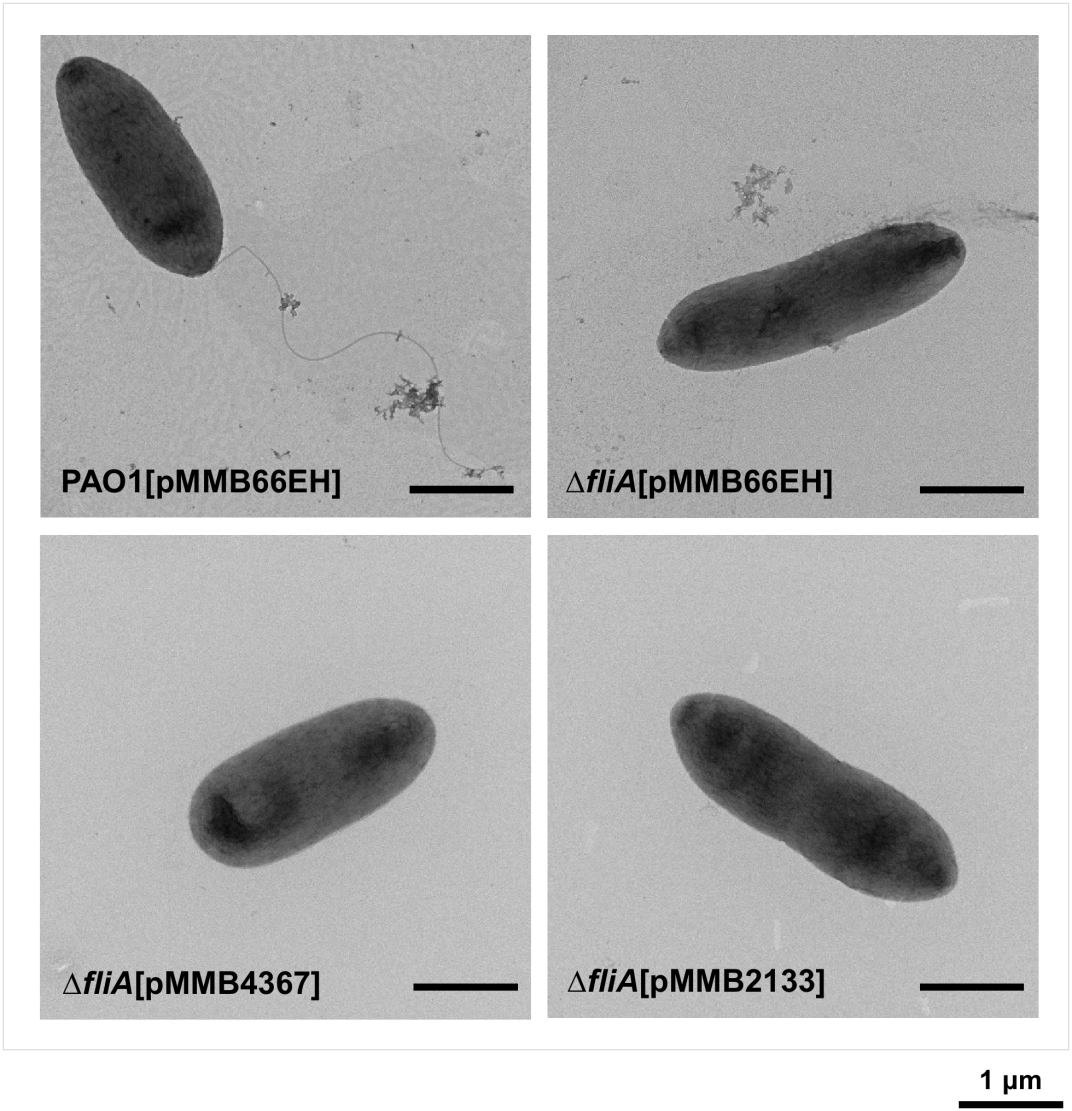
Effects of the c-di-GMP PDE coding genes *PA4367* and *PA2133* on flagellar synthesis in the *P*. *aeruginosa fliA* mutant. Cells from overnight cultures grown in LB broth supplemented with 500 μg/ml carbenicillin and 0.5 mM IPTG were examined through TEM.

Similar to the findings of the swarming assay, the Δ*fliA* mutant exhibited a defective phenotype in the swimming assay. However, in contrast to the results of the swarming assay, complementing the Δ*fliA* mutant with *PA4367* or *PA2133* failed to restore the defective swimming motility to normal (Fig 4). To elucidate the possible mechanisms underlying the difference in complementation, we examined the flagellar morphology of Δ*fliA* [pMMB66EH], Δ*fliA* [pMMB4367], and Δ*fliA* [pMMB2133] through transmission electron microscopy (TEM). The results revealed that the flagella were not only absent in Δ*fliA* [pMMB66EH] but also in Δ*fliA* [pMMB4367] and Δ*fliA* [pMMB2133] (Fig 6), suggesting that *fliA* regulates swarming motility through PDE-coding genes and is independent of flagellar biosynthesis.

## Discussion

C-di-GMP has been reported to be a regulator of bacterial motility [34–36]. Whether the secondary messenger crosstalks to the major motility transcription factor FliA is not clear. It has been shown that cellular c-di-GMP levels can affect expression of *Clostridium difficile sigD*, a *fliA* ortholog, and hence regulate toxin production in the bacterium [37]. On the other hand, FliA may regulate the expression of c-di-GMP metabolizing genes, as in the case of *E*. *coli yhjH* [11]. However, because of the presence of numerous c-di-GMP-metabolizing genes in a bacterium, less information exists on genes responsible for regulating the intracellular c-di-GMP concentration and hence the motility. This study demonstrated that FliA, in addition to transcriptionally controlling flagellar biosynthesis, regulated *P*. *aeruginosa* motility by modulating the c-di-GMP concentration. We identified the c-di-GMP-metabolizing genes responsible for regulating the c-di-GMP concentration in bacterial motility, and the result suggests that the *PA4367* gene is a target of FliA. Overall, these findings establish a basis for elucidating the mechanism underlying the FliA-mediated regulation of bacterial motility.

By using the RNAseq technology, we demonstrated that FliA regulates the expression of several PDE-encoding genes, including *PA4367*. However, moderate fold changes were observed in expression levels of the PDE-encoding genes between the wild-type and mutant Δ*fliA*. The GUS reporter gene assay revealed that *PA4367* is regulated by FliA. However, the involvement of additional PDEs in the FliA-mediated regulation of motility could not be eliminated. The presence of more than one PDE gene, with each contributing to a part of the c-di-GMP metabolism required in the bacterial motility regulation, may explain the slight fold changes in the expression of most PDE genes in the Δ*fliA* mutant.

TEM revealed that *PA4367* overexpression in the Δ*fliA* mutant did not restore the flagella (Fig 6); however, the defective swarming motility, and not the swimming motility, of the mutant could be rescued (Fig 4). The swimming motility of cells primarily depends on the flagella, whereas additional factors are involved in swarming motility [38,39]. Thus, under environmental conditions suitable for swimming, FliA may regulate bacterial motility through flagellar biosynthesis. When *P*. *aeruginosa* is cultivated on a solid surface, FliA may affect *PA4367* expression, which subsequently affects the c-di-GMP concentration and thus regulates swarming-associated factors, such as biosurfactants and type IV pili. Biosurfactants as wetting agents can reduce the surface tension required for swarming [39,40]. The hypothesis that *PA4367* overexpression results in increased pili production, which is associated with swarming [41], could not be confirmed through TEM, possibly because type IV pili are commonly present in a dynamic state and may not always be detected through TEM. Future studies examining the presence of type IV pili through Western blotting or fluorescence microscopy [42] may be required for verifying this hypothesis.

Complementation of the *fliA* mutant with pMMB*fliA*, a low copy number plasmid carrying a functional *fliA* gene, was conducted. The production of pyorubin and flagella biogenesis (examined under electron microscopy) could be restored partially in the complementation strain, indicating that *fliA* is responsible for these two phenotypes. However, the swimming and swarming phenotype of the *fliA* mutant could not be restored by complementation. The finding is not surprising because that FliA is known to involve in complex regulation of flagellar biogenesis in *P*. *aeruginosa* [43,22,44]. One possible explanation is that excessive FliA leads to feedback inhibition through activating expression of *flgM* which encodes an anti-FliA activity. Therefore an optimal quantity of FliA is critical to the bacterial motility.

In *E*. *coli*, bacterial motility could be regulated by PDE YhjH and YcgR. The mechanism underlying the regulated expression of *yhjH* in *E*.*coli* remains unclear. The YcgR ortholog could be unambiguously identified in *P*. *aeruginosa* PAO1. We previously constructed an isogenic mutant of YcgR in *P*. *aeruginosa* PAO1, and its phenotype was consistent with that reported for *E*. *coli* YcgR.[9] By contrast, the corresponding gene of *E*. *coli yhjH* in *P*. *aeruginosa* PAO1 remains unclear. Restoring the defective swarming motility of *P*. *aeruginosa* Δ*fliA* by complementing it with *PA4367* (Fig 4) suggests that this gene plays a functional role equivalent to that of *yhjH* in *E*. *coli* [9,10]. Finally, both RNAseq and GUS reporter assay confirmed that *PA4367* is a downstream target of FliA in *P*. *aeruginosa* PAO1 (Table 2). This finding is also consistent with that of a previous study stating that FliA regulates *yhjH* in *E*. *coli* [26].
